# Correction: TNF-α Induces Cytosolic Phospholipase A_2_ Expression in Human Lung Epithelial Cells via JNK1/2- and p38 MAPK-Dependent AP-1 Activation

**DOI:** 10.1371/annotation/f56711b9-78f1-49ed-9116-872913e98867

**Published:** 2013-12-19

**Authors:** I-Ta Lee, Chih-Chung Lin, Shin-Ei Cheng, Li-Der Hsiao, Yu-Chun Hsiao, Chuen-Mao Yang

In Figure 2, section A, the Western blot image of GRPDH was incorrecly posted as a duplicate of Act. D. Please view the corrected Figure 2A here: 

**Figure:**
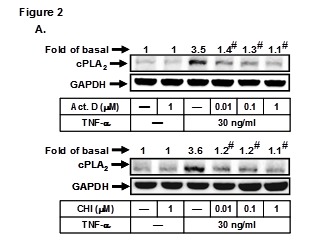


In Figure 6, section C, the Western blot image of GAPDH in the transfection fith c-fos siRNA was incorrectly posted as a duplicate of c-jun siRNA. Please view the corrected Figure 6C here: 

**Figure:**
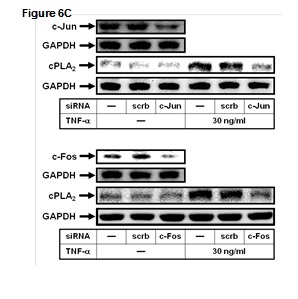


The affiliation for the Editor should read, "Guangwei Liu, Shanghai Medical College, Fudan University, Shanghai, China 

